# DNaseI Protects against Paraquat-Induced Acute Lung Injury and Pulmonary Fibrosis Mediated by Mitochondrial DNA

**DOI:** 10.1155/2015/386952

**Published:** 2015-02-11

**Authors:** Guo Li, Li Yuzhen, Chen Yi, Chen Xiaoxiang, Zhou Wei, Zhu Changqing, Ye Shuang

**Affiliations:** ^1^Department of Rheumatology, South Campus, Ren Ji Hospital, School of Medicine, Shanghai Jiao Tong University, Shanghai 200001, China; ^2^Department of Emergency Medicine, Ren Ji Hospital, School of Medicine, Shanghai Jiao Tong University, Shanghai 200001, China

## Abstract

*Background*. Paraquat (PQ) poisoning is a lethal toxicological challenge that served as a disease model of acute lung injury and pulmonary fibrosis, but the mechanism is undetermined and no effective treatment has been discovered. *Methods and Findings*. We demonstrated that PQ injures mitochondria and leads to mtDNA release. The mtDNA mediated PBMC recruitment and stimulated the alveolar epithelial cell production of TGF-*β*1 in vitro. The levels of mtDNA in circulation and bronchial alveolar lavage fluid (BALF) were elevated in a mouse of PQ-induced lung injury. DNaseI could protect PQ-induced lung injury and significantly improved survival. Acute lung injury markers, such as TNF*α*, IL-1*β*, and IL-6, and marker of fibrosis, collagen I, were downregulated in parallel with the elimination of mtDNA by DNaseI. These data indicate a possible mechanism for PQ-induced, mtDNA-mediated lung injury, which may be shared by other causes of lung injury, as suggested by the same protective effect of DNaseI in bleomycin-induced lung injury model. Interestingly, increased mtDNA in the BALF of patients with amyopathic dermatomyositis-interstitial lung disease can be appreciated. *Conclusions*. DNaseI targeting mtDNA may be a promising approach for the treatment of PQ-induced acute lung injury and pulmonary fibrosis that merits fast tracking through clinical trials.

## 1. Introduction

Acute lung injury and subsequent pulmonary fibrosis are a common clinically critical problem with extremely high mortality and morbidity. Multiple reasons, such as environmental factors, infections, and autoimmune factors, can trigger or perpetuate this pathophysiological process of aberrant lung injury and healing, but its precise mechanism is largely undetermined. Currently, no effective treatment has been shown to halt or reverse its development or progression [1].

Paraquat (1,1-dimethyl-4,4-bipyridinium dichloride, PQ) is one of the most widely used nonselective bipyridyl herbicides around the world, particularly in developing countries, such as China and India. PQ poisoning is a lethal toxicological challenge to humans and animals and is characterized by acute lung injury and irreversible pulmonary fibrosis [2–5]. No known antidote for PQ has been discovered, and the treatment options are merely supportive [6]. Thus, exploring the mechanism of PQ-induced lung injury may help to develop life-saving treatments for PQ poisoning and serve as a unique disease model for studying other types of acute lung injury and pulmonary fibrosis.

It has been demonstrated that PQ is toxic to green plants by attacking chloroplasts and interfering with vital photosynthesis [7]. In mammals, mitochondria are the counterpart organelle to chloroplasts. PQ cytotoxicity as a result of targeting mitochondria was proposed and demonstrated both in vitro and in vivo several decades ago [8–10], but the exact mechanism of PQ-induced mitochondrial dysfunction and, more importantly, how this links to lung injury and fibrosis are unknown. It is noteworthy that PQ cannot be detected in the plasma a few hours after ingestion due to rapid clearance by the kidneys [11]. Therefore, a PQ-triggered but self-sufficient pathway that mediates the subsequent lung injury and fibrosis is likely. Mitochondria are endosymbionts that originated from purple bacteria approximately 1.5 × 10^9^ years ago [12] and are the only organelle of the mammalian cell except for the nucleus that contains its own DNA, that is, mitochondrial DNA (mtDNA) [13]. mtDNA contains a higher frequency of unmethylated cytosine-phosphate-guanine (CpG) dinucleotides, similar to bacterial DNA, which can promote innate immune responses through TLR-9 [14, 15]. Circulating mtDNA from tissue injury was recently found to be essential to mediate the systemic inflammatory response and target organ damage [16]. Mitochondria have thus been termed the “Trojan Horse,” capable of triggering inflammation and forming a vicious circle that results in profound tissue injury [17].

This study attempted to identify pathways underlying the role of PQ in mitochondrial dysfunction and mtDNA release in lung injury. The results may herald new interventions for treating this fatal toxicological condition. In addition, they may also suggest new methods for the management of other forms of acute lung injury and pulmonary fibrosis.

## 2. Methods

### 2.1. Reagents and Cell Lines

Paraquat and DNaseI were purchased from Sigma (St. Louis, MO, USA). Human alveolar type II-like epithelial A549 cells, human pulmonary fibroblast (HFL1) cells, and human pulmonary artery endothelial cells (HPAECs) were obtained from Cell Bank (Shanghai Institute of Cell Biology, China). The culture conditions were adapted from a previous report [18]. The Cell Counting Kit-8 (Dojindo, Japan), the mitochondrial-specific cationic dye JC-1 (Molecular Probes, OR, USA), human IL-1*β*, IL-6, TNF-*α*, and TGF-*β*1 ELISA kits (R&D, Minneapolis, MN, USA), and the Vascular Permeability Kit (Millipore Corporation) were all used according to the manufacturers' protocols.

### 2.2. Measurement of mtDNA and Real-Time PCR

A549 cells were seeded into sterile, flat-bottom, 6-well plates at 0.5 × 10^6^ cells/well and grown overnight to 80% confluence. They were challenged with a control or various concentrations of PQ. Cell supernatants were harvested and centrifuged at 800 rpm for 5 min to remove cellular debris. mtDNA was extracted from the supernatants using a QIAamp DNA Blood Mini Kit (Qiagen, USA). The concentration of mtDNA was determined using a standard curve generated by quantitative PCR (qPCR) (construct plasmids (PGM-T) containing human or mouse mitochondrial CytoB gene sequences; the primers are provided in Table 1). Total RNA was extracted using TRIzol reagent (Invitrogen, USA) and reverse-transcribed using the Prime Script cDNA Synthesis Kit (Takara, Japan). The primers for *α*-SMA, type I collagen, type III collagen, vimentin, N-cadherin, E-cadherin, TJP-1, cytokeratin, and *β*-actin are also provided in Table 1. qPCR was performed using SYBR Premix Ex (Takara, Japan) on an ABI Prism 7900HT system.

### 2.3. Chemotaxis Assays and Flow Cytometry

PQ-primed A549 cell supernatants enriched for mtDNA (approximately 10^4^ copies/*μ*L) were treated with or without DNaseI for the chemotaxis assay. Whole blood samples were obtained from healthy volunteers. Cells were isolated immediately by a one-step gradient centrifugation method using Polymorphprep reagent according to the described protocol [19]. The chemotactic responses of PBMCs and PMNs were assessed by transwell cell culture chambers with polycarbonate filters with 5 *μ*m pores. The fold chemotaxis index was calculated by dividing the number of cells migrating in the presence of supernatants by those migrating toward medium alone. Anti-CD14-APC, anti-CD3-FITC, and anti-CD19-PE (BD Biosciences, San Jose, CA) were used to distinguish the subtypes of the migrated cells by flow cytometry.

### 2.4. Real-Time Cell Analysis

HPAECs or HFL1 cells were seeded into 96-well microtiter plates (E plate) at a density of 5000 cells/well. After cell synchronization, the cells were treated with different stimuli and monitored. Transendothelial monolayer electrical resistance [20] and the cell index were measured using the xCELLigence Real-Time Cell Analyzer (Roche, USA).

### 2.5. Animals and Study Protocols

C57BL/6J mice (8–10 weeks of age, *n* = 144), all males, were purchased from the Shanghai SLAC Laboratory Animal Co. Ltd. (Shanghai, China). The mice were intraperitoneally injected with PQ (40 mg/kg or 25 mg/kg, as indicated) or normal saline on day 1. Some mice were injected intravenously with DNaseI (0.3 mg/kg, 3 mg/kg, or 30 mg/kg) or vehicle on day 0 (one day before the PQ or sham exposure), day 2, day 5, and day 8. Before euthanasia, the mice were anesthetized with pentobarbital (60 mg/kg) and subsequently underwent a median thoracosternotomy, bronchoalveolar lavage, exsanguination via the inferior vena cava, and removal of both lungs. The left lung was cut into two pieces, snap-frozen in liquid nitrogen, and stored at −80°C until mRNA extraction. The right lung was immediately fixed in 10% neutral buffered formalin and embedded in paraffin for hematoxylin and eosin staining (HE) or Masson's trichrome staining. Survival experiments (for each group, *n* = 10) were repeated three times. Survival curves were calculated by pooling the data of the three independent experiments. The bleomycin-induced lung injury model was generated according to a previous report [21]. Survival experiment was also carried out accordingly. A quantitative fibrosis scale (Ashcroft scale) was used [22]. Immunohistochemical staining for *α*-SMA was performed according to the manufacturer's instructions. The study protocols were approved by the Animal Care Committees of Shanghai Jiao Tong University School of Medicine.

### 2.6. BALF from Patients with Amyopathic Dermatomyositis-Related Interstitial Lung Disease (CADM-ILD) or Controls

BALF was obtained from patients with CADM-ILD (*n* = 14) or patients with pulmonary solitary mass or nodule (*n* = 11) as controls. All patients were following a standardized bronchoscope procedure, and controls' BALF was recovered from the contralateral (“normal”) side of the middle lobe. The study protocol was approved by the institutional review board of Shanghai Renji Hospital, Shanghai Jiao Tong University School of Medicine, with patients' informed consent obtained.

### 2.7. Statistical Analysis

The data are shown as the mean ± SEM of at least three independent experiments. The statistical significance between groups was analyzed using GraphPad Prism v4.0 Software (San Diego, CA, USA). Two-group comparisons of continuous data were assessed by either a two-tailed Student's *t*-test or a nonparametric Mann-Whitney *U* test. ANOVA with a Bonferroni correction was used for multiple comparisons. Kaplan-Meier survival curves were calculated. A *P* value less than 0.05 was considered significant.

## 3. Results

### 3.1. PQ Injures Mitochondria and Leads to mtDNA Release

**Figure 1:**
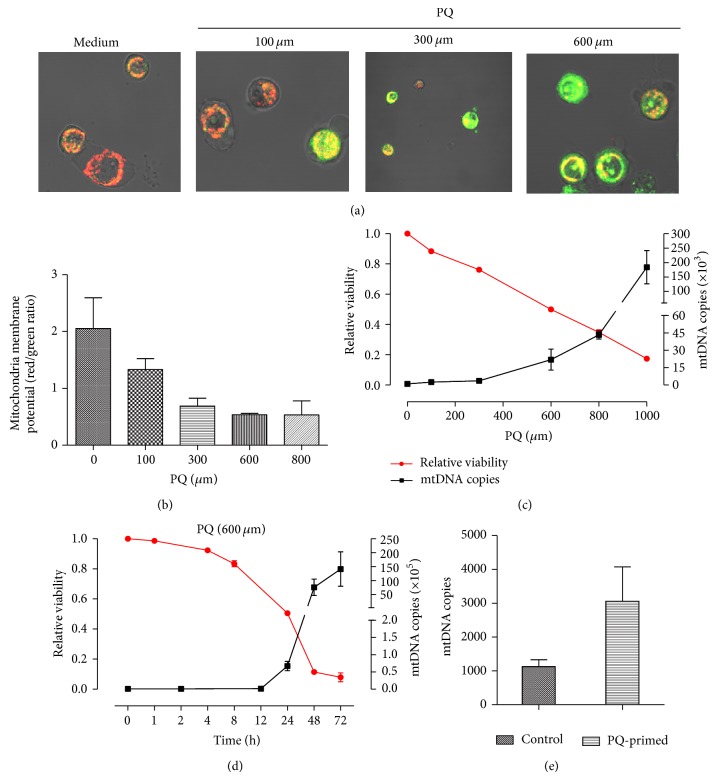
PQ injures mitochondria and leads to mtDNA release. ((a)-(b)) The mitochondrial membrane potential decreased in A549 cells (changed from red to green) following a 24 h exposure to various doses of PQ. PQ reduced A549 cell viability (red line) in a dose- (c) and time-dependent manner ((d), PQ 600 *μ*m) and was correlated with increased mtDNA release (black line) into the supernatant. (e) A549 cells were incubated with or without (control) 600 *μ*m PQ for 12 h and then washed. Fresh medium was added, and the cells were cultured for 12 h. The supernatant mtDNA level was elevated among PQ-primed cells.

We first assessed the impact of PQ on mitochondria in human alveolar type II-like epithelial A549 cells by fluorescence staining with the mitochondrial membrane potential-dependent dye JC-1. The mitochondrial membrane potential was decreased in the presence of PQ in a concentration-dependent manner (Figures 1(a) and 1(b)), indicating that the integrity of the mitochondria was disrupted by PQ. A similar result was obtained in a CCK8 assay, which demonstrated a reduction of cell viability and increased mtDNA release into the cell supernatant in a PQ dose- and time-dependent manner (Figures 1(c) and 1(d)). To further mimic the rapid clearance of PQ in vivo, cells were treated with PQ for 12 h (PQ-primed) and then washed. Fresh medium was added, and the cells were incubated for 12 h. mtDNA release into the supernatant continued after PQ removal (Figure 1(e)), which is consistent with an ongoing injury response. PQ-primed supernatants enriched with mtDNA were used in the following secondary cultures.

### 3.2. PQ-Induced mtDNA Release Can Enhance PBMC Recruitment

**Figure 2:**
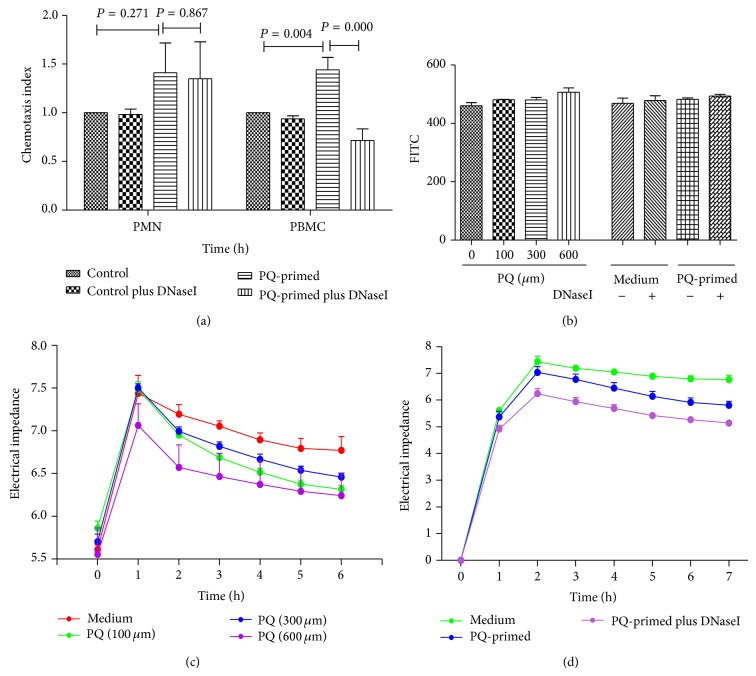
PQ-induced mtDNA release can enhance PBMC recruitment. (a) The chemotactic index of PBMCs but not PMNs increased after incubation with PQ-primed mtDNA-enriched supernatant. (b) The endothelial transwell fluorescein leakage assay displayed no difference in the presence of either PQ or PQ-primed mtDNA. ((c)-(d)) PQ and PQ-primed mtDNA had no statistical significant impact on endothelial cell integrity, as assessed with transendothelial monolayer electrical resistance.

PQ-induced acute lung injury is characterized by inflammatory responses. We therefore investigated whether PQ-primed mtDNA could recruit effector cells. Indeed, enhanced chemotaxis of PBMCs but not neutrophils was observed under PQ-primed mtDNA-enriched conditions in a transwell assay; the effect was abolished when DNaseI was added to remove the mtDNA (Figure 2(a)). Flow cytometry analysis showed no difference in chemotaxis between PBMC subtypes, including T cells, B cells, NK cells, and monocytes (data not shown). This is likely to be an active attraction process instead of passive leaking due to the breakdown of the endothelial barrier. There was no significant change in transendothelial permeability as measured using transendothelial monolayer electrical resistance and an endothelial transwell fluorescein leakage assay, regardless of the presence of PQ or PQ-primed mtDNA-containing supernatants (Figures 2(b), 2(c), and 2(d)).

### 3.3. mtDNA Stimulates Alveolar Epithelial Cell Production of TGF-*β*1

**Figure 3:**
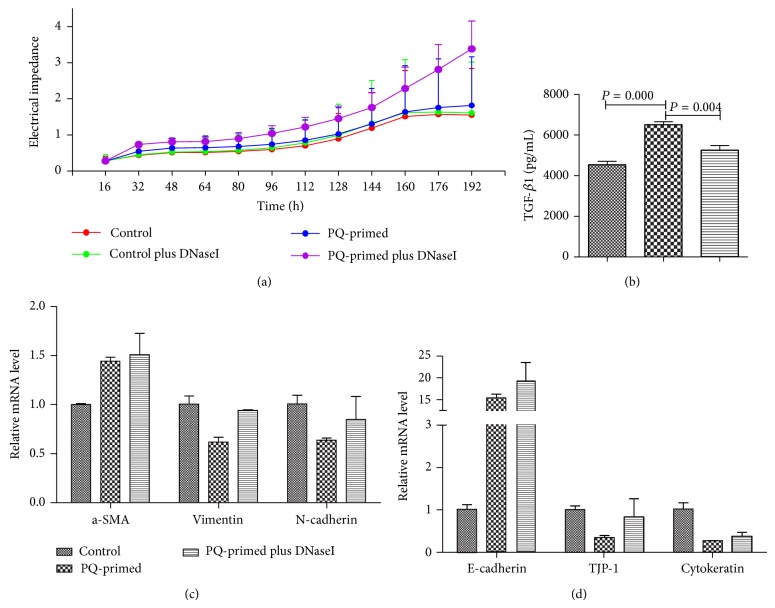
PQ-primed mtDNA stimulates alveolar epithelial (A549) cell production of TGF-*β*1. (a) PQ-primed mtDNA did not stimulate fibroblast (HFL1) proliferation. (b) PQ-primed mtDNA increased A549 cell TGF-*β*1 expression but had no effect on epithelial-mesenchymal transition marker expression (c) nor on downregulating epithelial marker expression (d).

We next evaluated how PQ-induced pulmonary fibrosis was mediated by mtDNA. We initially investigated human pulmonary fibroblast (HFL1) cells and observed no direct proproliferation effect of PQ-primed mtDNA on the fibroblasts (Figure 3(a)). It was also possible that the recruited circulating monocytes could be precursors that transform into fibrocytes [23]. However, the impact of this transformation could not be observed in the presence of mtDNA or PQ in a conditioned culture of mouse splenocytes (data not shown). Epithelial-mesenchymal transition may also occur and be essential in pulmonary fibrosis. Indeed, PQ-primed mtDNA stimulated alveolar epithelial cells (A549) to produce TGF-*β*1 (Figure 3(b)), although the epithelial-mesenchymal transition markers *α*-SMA, vimentin, and N-cadherin were not increased. Likewise, real-time PCR demonstrated that the epithelial markers E-cadherin, TJP-1, and cytokeratin were not downregulated (Figures 3(c) and 3(d)). Quite opposite, E-cadherin was upregulated by PQ for unknown reason, and this effect is apparently independent of mtDNA. Nevertheless, it is conceivable that TGF-*β*1 acts as a critical factor driving fibrosis and is upregulated by PQ-primed mtDNA to further act in the fibrogenic pathway in PQ-induced pulmonary fibrosis.

### 3.4. Circulating and BALF mtDNA Are Elevated in a Mouse Model of PQ-Induced Lung Injury

**Figure 4:**
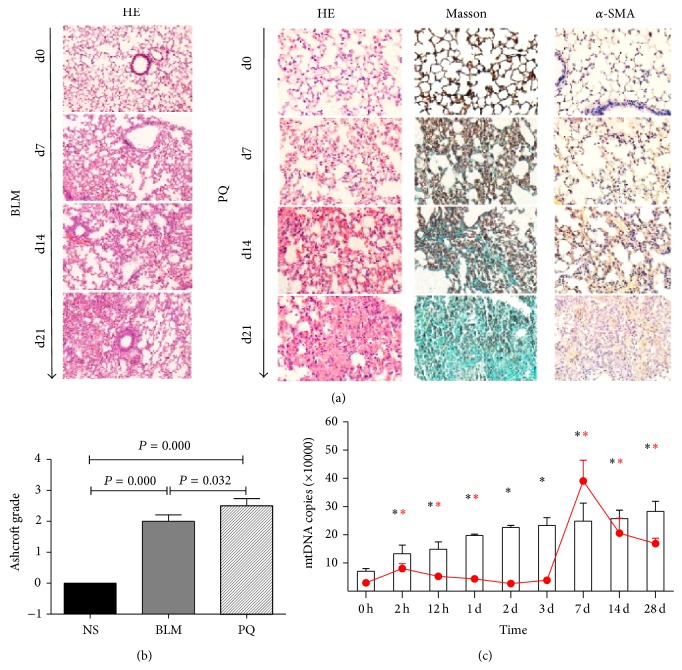
The levels of circulating and BALF mtDNA are elevated in a mouse model of PQ-induced lung injury. ((a)-(b)) C57BL/6 mice injected with PQ (25 mg/kg, i.p., d1 and d3) displayed typical acute lung injury and pulmonary fibrosis compatible with the classic bleomycin (BLM) model. (c) The time course of mtDNA detection differed in the plasma (bar) and BALF (red line).

A PQ-induced lung injury model was established (25 mg/kg PQ intraperitoneally injected on day 1 and day 3). The model resulted in better homogeneity in lung pathology compared to the classic bleomycin (BLM) model, which is intratracheally delivered (Figures 4(a) and 4(b)). The mtDNA level was elevated in the plasma and BALF of mice administered PQ, but the patterns differed between the two (Figure 4(c)). The mtDNA level peaked in the BALF at day 7, which is more representative of acute lung injury that subsequently induces pulmonary fibrosis.

### 3.5. DNaseI Protects PQ or Bleomycin-Induced Lung Injury in Mice and Improves Survival

**Figure 5:**
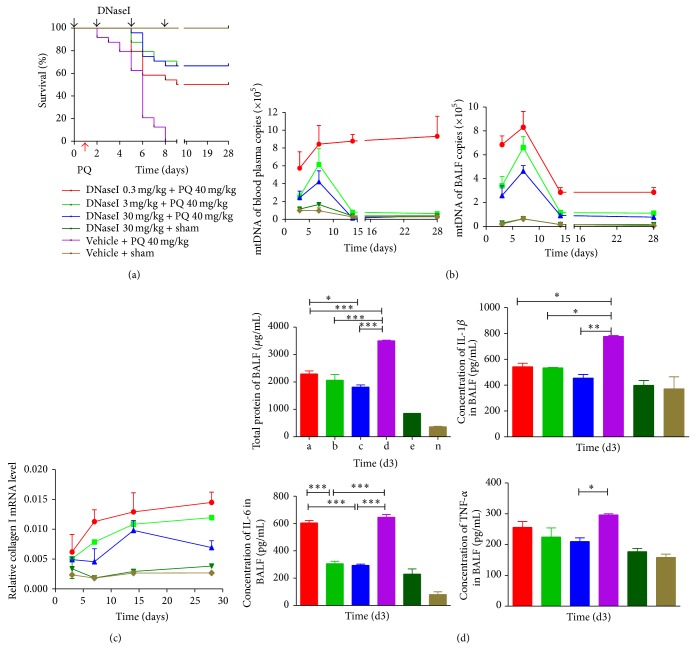
DNaseI prevents PQ-induced lung injury in mice and improves survival. (a) C57BL/6 mice (*n* = 30 for each group) were challenged with an acute lethal dose of PQ (40 mg/kg, i.p.) or sham exposure on day 1. Some mice were administered DNaseI or vehicle at the indicated doses on days 0, 2, 5, and 8. (b) DNaseI suppressed the circulating and BALF mtDNA levels in a dose-dependent manner. ((c)-(d)) Downregulation of total protein exudation, TNF*α*, IL-1*β*, IL-6, and collagen I in a DNaseI dose-dependent manner.

**Figure 6:**
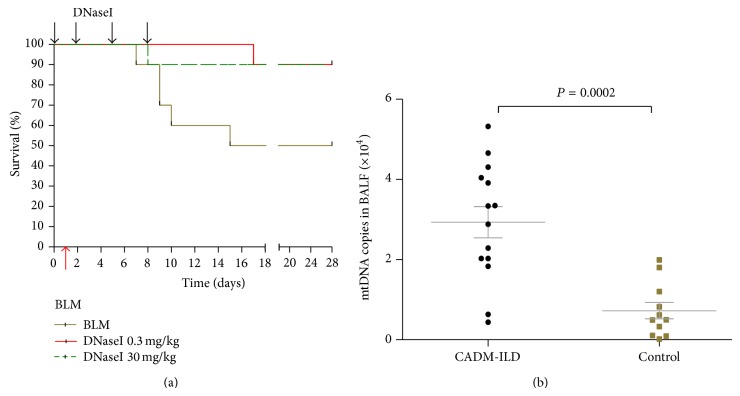
DNaseI improves survival in bleomycin-induced pulmonary fibrosis mice and mtDNA was increased in BALF from CADM-ILD patients. (a) Improved survival in bleomycin-induced pulmonary fibrosis mice by i.v. administration of DNaseI (*n* = 10 for each group). (b) mtDNA in BALF from patients with CADM-ILD (*n* = 14) was significantly elevated compared to controls (*n* = 11). Mean and SEM values were indicated.

DNaseI was administered intravenously 1 day prior to (day 0) exposure to the lethal dose of PQ (40 mg/kg, i.p.) and on days 2, 5, and 8 after the PQ challenge. DNaseI resulted in significant protection against PQ poisoning, as demonstrated by up to 70% survival observed in the DNaseI treatment group compared to the 100% mortality observed in the PQ control (Figure 5(a)). BALF and circulating mtDNA were eliminated in vivo by DNaseI (Figure 5(b)) in parallel with the downregulation of acute lung injury markers, including BALF total protein exudation, TNF*α*, IL-1*β*, and IL-6, at day 3 and marker of fibrosis, collagen I, at day 28 in a DNaseI dose-dependent manner (Figures 5(c) and 5(d)). The improved survival was also appreciated in the classic bleomycin-induced pulmonary fibrosis mice model. 90% survival was achieved in the DNaseI treatment groups versus a 50% survival in the bleomycin control group on day 28 (Figure 6(a)).

### 3.6. mtDNA Were Increased in BALF from Patients with CADM-ILD

mtDNA in BALF from patients with CADM-ILD was significantly elevated compared to controls (*P* = 0.0002) (Figure 6(b)).

## 4. Discussion

In the current study, we demonstrated that PQ injures mitochondria, causing subsequent mtDNA release. During PQ-induced oxidative stress, mitochondria can be a major source of reactive oxygen species (ROS) production [24–27]. mtDNA, located close to the inner mitochondrial membrane where ROS are generated, is susceptible to oxidative damage [28]. In addition, ROS may also facilitate mtDNA release [29]. However, the mechanism by which PQ targets mitochondria is still undetermined and requires further investigation. As an example, Chen and colleagues observed that PQ-induced Nrf-2, an antioxidative transcriptional factor, in lung alveolar epithelial cells and Nrf-2 siRNA reversed the PQ-induced mRNA expression profile in vitro [30]. Paradoxically, in vivo data suggested that Nrf2^−/−^ mice are more susceptible to gastric aspiration-induced acute lung injury [31], as well as to* Staphylococcus aureus*-induced lung injury [32]. Nevertheless, our data are the first to demonstrate that mtDNA is the mediator of PQ-induced acute lung injury and pulmonary fibrosis. mtDNA is a double-stranded, closed circular molecule of 16,569 nucleotide pairs. As a potent innate immune system stimulator, Zhang and colleagues found that circulating mtDNA and formyl peptides can induce a systemic inflammatory response to injury via TLR9 and other DAMP pattern recognition pathways' activation [16]. According to our data, PQ-induced mtDNA is capable of mediating the recruitment of mononuclear cells but not polymorphonuclear cells. This chemotaxis process is apparently independent of endothelial barrier disruption. PQ-induced mtDNA can also enhance alveolar epithelial cell TGF-*β*1 production. As one of the key profibrotic molecules, TGF-*β*1 is elevated in the lung tissue of PQ treated mice according to our previous data [33], similar to the finding in bleomycin model [34]. More importantly, the effects of mtDNA in vitro are all reversible in the presence of DNaseI.

The most interesting finding was the in vivo observation that the intravenous administration of DNaseI displayed a striking protective effect against both PQ- and bleomycin-induced lung injury in mice. It is noteworthy that early intervention with DNaseI in the acute lung injury phase is crucial and parallels the timing of the BALF mtDNA peak in PQ model. In addition to its wide use in molecular biology research, DNaseI has been approved as a nebulizing agent for patients with cystic fibrosis [35]. Endogenous DNaseI deficiency due to a genetic polymorphism in lupus has been postulated to be responsible for inappropriate nuclear debris clearance and results in immune system overload and antinuclear autoantibody generation [36, 37]. However, a phase I trial in SLE patients failed to display efficacy of the intravenous and subcutaneous administration of recombinant human DNaseI, although its safety profile was acceptable [38].

Caudrillier and colleagues recently found that the formation of neutrophil extracellular traps (NETs) is essential to mediate transfusion-related acute lung injury [39]. DNaseI and histone-blocking antibodies that inhibit NET formation are both protective against transfusion-related acute lung injury in mice. Although the pathogenesis of this acute lung injury is different from PQ-induced lung injury, a shared pathway may exist. For example, the possibility that mtDNA is an important NET component cannot be excluded. It is also informative that the mtDNA level is significantly elevated in the BALF of patients with CADM-ILD, which is another lethal autoimmune condition with acute lung injury and pulmonary fibrosis [40]. There are increasing evidences suggesting that mtDNA is a promising therapeutic target, such as mtDNA repair enzyme 8-oxoguanine DNA glycosylase 1 and DNA repair enzyme endonuclease III which all displayed a certain protective effect against different models of lung injury [41–44]. Taken together, the rationale for initiating clinical trials on DNaseI for PQ-induced lung injury is sound, and such attempts to tame the “Trojan Horse” in other forms of lung injury and pulmonary fibrosis will be breathtaking.

## Figures and Tables

**Table 1:** Primers used in the experiment.

Genes	Forward primer	Reverse primer
GAPDH (human)	5′-GCACCGTCAAGGCTGAGAAC-3′	5′-ATGGTGGTGAAGACGCCAGT-3′
Cytochrome B (human)	5′-ATGACCCCAATACGCAAAAT-3′	5′-CGAAGTTTCATCATGCGGAG-3′
*α*-SMA (human)	5′-GCGTGGCTATTCCTTCGTTACT-3′	5′-GCTACATAACACAGTTTCTCCTTGATG-3′
Vimentin (human)	5′-CCGCCCTAGACGAACTGGGTC-3′	5′-AGGCTGTGGACAGTGGCTTCTG-3′
N-cadherin (human)	5′-CCTCCAGAGTTTACTGCCATGAC-3′	5′-GTAGGATCTCCGCCACTGATTC-3′
E-cadherin (human)	5′-GCCTCCTGAAAAGAGAGTGGAAG-3′	5′-TGGCAGTGTCTCTCCAAATCCG-3′
Tjp-1 (human)	5′-GTCCAGAATCTCGGAAAAGTGCC-3′	5′-CTTTCAGCGCACCATACCAACC-3′
Cytokeratin (human)	5′-AGCAGAATCGGAAGGACGCTGA-3′	5′-ACCTCGCTCTTGCTGGACTGAA-3′
*β*-actin (mice)	5′-ATGCTCCCCGGGCTGTAT-3′	5′-CATAGGAGTCCTTCTGACCCATT-3′
Cytochrome B (mice)	5′-CCTATCAGCCATCCCATAT-3′	5′-GGAAGAGGAGGTGAACGA-3′

## Abbreviations

PQ: Paraquat
mtDNA: Mitochondrial DNA.
